# β-Catenin: oncogenic role and therapeutic target in cervical cancer

**DOI:** 10.1186/s40659-020-00301-7

**Published:** 2020-08-05

**Authors:** Bingqi Wang, Xianping Li, Lei Liu, Min Wang

**Affiliations:** Department of Laboratory Medicine, The Second Xiangya Hospital, Central South University, Changsha, 410011 Hunan China

**Keywords:** β-Catenin, Cervical cancer, Cell–cell adhesion, Wnt signaling pathway

## Abstract

Cervical cancer is a common and fatal malignancy of the female reproductive system. Human papillomavirus (HPV) is the primary causal agent for cervical cancer, but HPV infection alone is insufficient to cause the disease. Actually, most HPV infections are sub-clinical and cleared spontaneously by the host immune system; very few persist and eventually develop into cervical cancer. Therefore, other host or environmental alterations could also contribute to the malignant phenotype. One of the candidate co-factors is the β-catenin protein, a pivotal component of the Wnt/β-catenin signaling pathway. β-Catenin mainly implicates two major cellular activities: cell–cell adhesion and signal transduction. Recent studies have indicated that an imbalance in the structural and signaling properties of β-catenin leads to various cancers, such as cervical cancer. In this review, we will systematically summarize the role of β-catenin in cervical cancer and provide new insights into therapeutic strategies.

## Background

Cervical cancer is among the most common female malignant tumors. In 2018, cervical cancer was the fourth diagnosed malignancy and the fourth leading cause of cancer death in women worldwide. With the development of precancerous screening methods and popularization of Human papillomavirus (HPV) vaccination programs, the morbidity and mortality of cervical cancer in high-income countries have declined. However, in low- and middle-income countries, cervical cancer continues to be a heavy burden and threatens female health [1]. Hence, a fundamental study of the molecular mechanism of cervical cancer remains imperative.

HPV has been identified as a necessary cause of cervical cancer. Based on epidemiological studies, the viral infection rate might amount to 80% in sexually active women [2]. A vital step during HPV infection is integrating viral genes into the host chromosomal DNA, which leads to unusual and uncontrolled cell growth, differentiation, and death [3]. Fortunately, most HPV infections are self-limited and cleared by the immune system within several months. Only a few people can develop persistent HPV infection and progress to cervical cancer [4]. So, exposure to HPV alone is “necessary but not sufficient” for cancer carcinogenesis; other factors, such as genetics, epigenetics, environment, immunity, and behavior, may also impact [5].

β-Catenin, the vertebrate homolog of Drosophila Armadillo, is a multifunction protein encoded by the *CTNNB1* gene in humans [6]. As a member of the catenin protein family, it has a significant role in regulating and coordinating intracellular adhesion. As a critical regulator of the Wnt signaling pathway, which controls embryonic development and adult tissue homeostasis, β-catenin functions as a transcriptional activator when coupled with members of the T cell factor/lymphoid enhancer factor (TCF/LEF) family of DNA-binding proteins [7]. In normal epithelial cells, the level of cytoplasmic β-catenin is regulated, and membrane localization is observed. While in multiple cancer cells, including breast cancer [8], gastric cancer [9], bone cancer [10], colorectal cancer [11], and hepatocellular carcinoma [12], the cytosolic β-catenin elevates for the mutations of Wnt/β-catenin pathway core components. To date, accumulating evidence has confirmed that cervical cancer is attributed to the imbalance in the structural and signaling properties of β-catenin [5]. Yet, most pioneering work has not systematically explained how β-catenin takes part in cervical cancer. Therefore, this review will center on the growing evidence implicating in the function, regulation, and alteration of β-catenin in cervical neoplasia, to provide theoretical insights for cervical cancer prevention and therapy.

## Main text

### β-Catenin: structure and subcellular localization

**Fig. 1:**
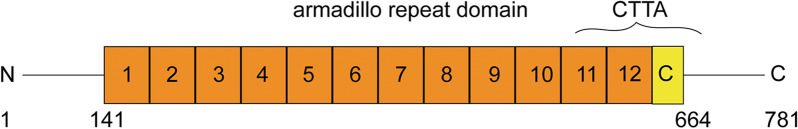
The structure of β-catenin. β-Catenin has a central armadillo repeat domain (residues 141-664) composed of 12 armadillo repeats, flanked by well-defined amino terminus domain (NTD) and carboxyl terminus domain (CTD)

β-Catenin is a 781-amino-acid protein and belongs to the armadillo protein families. It was initially discovered in the late 1980s as an E-cadherin-associated protein [13] and characterized in primary screening of genes required for embryonic development in Drosophila [14]. The central region of β-catenin includes 12 Armadillo (ARM) repeats, flanked by the well-defined amino-terminus domain (NTD) and carboxyl terminus domain (CTD) [15]. Between the last ARM repeat and the flexible part of the CTD is a specific conserved helix, which was essential for the signaling activity of β-catenin [16]. Each “ARM repeat” comprises a repeating 42 amino acid motif and forms three helices in a triangular shape. All “ARM repeats” form a superhelix that features a long and positively charged groove [17]. Using the core ARM domain structure, the central region of β-catenin forms a scaffold and provides an interactive platform for β-catenin binding partners. Most binding partners share overlapping binding sites, while β-catenin interacts with them in a mutually exclusive fashion. One possibility is that spatial segregation of different binding partners exerts a role in regulating its binding properties. Another explanation could be the competition among them, which is indispensable for enabling the functions of these proteins. Conformation changes in β-catenin may also be helpful [18]. Altogether, the distinct features of β-catenin depend on the structural composition of β-catenin (Fig. 1).

Cellular β-catenin exists in three different pools: membranous, cytoplasmic, and nuclear [19]. Freshly synthesized β-catenin interacts with E-cadherin and serves as a structural protein localized to the cell membrane (membranous pool). In contrast, destruction complex captures free β-catenin in the cytoplasm and rapidly targets it for degradation (cytoplasmic pool). Due to the compromised function of the destruction complex, β-catenin escapes degradation and translocates into the nucleus (nuclear pool) [19]. In different cell pools, β-catenin undergoes distinct regulation patterns and have diverse functions.

### Dual functions of β-catenin

β-Catenin executes two main tasks: structural role in the adherens junctions and signaling activity in the gene transcription. These two functions are orchestrated by mechanisms, which regulate the spatial separation, retention, or stability of β-catenin.

#### β-Catenin: intracellular adhesion regulator

**Fig. 2:**
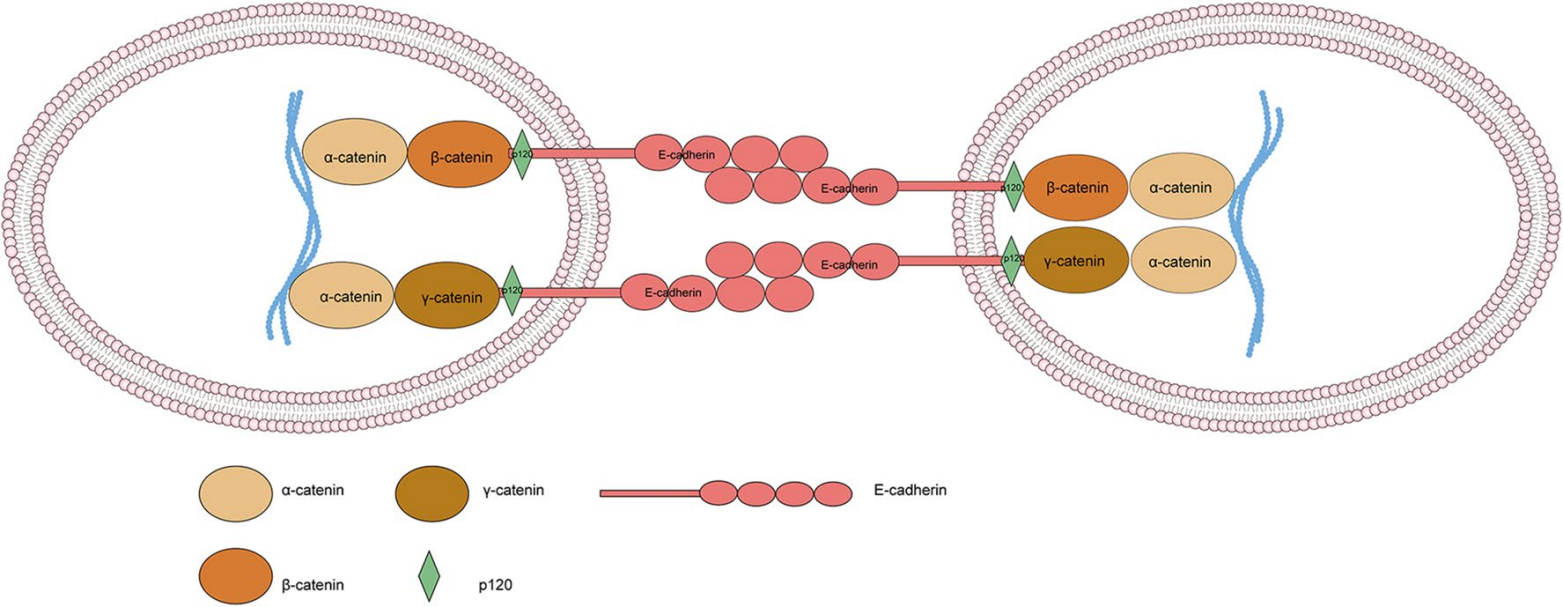
E-Cadherin/β-catenin complex. This complex is a crucial element in cell–cell adhesion; it consists of three proteins: E-cadherin, β-catenin, and α-catenin. E-Cadherin immobilizes newly synthesized β-catenin to the cell membrane and α-catenin links β-catenin to the actin cytoskeleton

Cell–cell junctions are requisite for maintaining cell and tissue polarity and integrity. One of the cancer characteristics is defective cell–cell and cell–matrix adhesion [20]. Vertebrates comprise three intercellular junction systems: tight junction, adhesion junction, and gap junction. Among constituent structural molecules that assemble to form adhesion junctions, cadherin/catenin-based anchoring junctions organize and tether microfilaments to maintain cell adhesive properties and integrate intra- and intercellular signaling [21]. Classical cadherins are single-pass transmembrane glycoproteins mediating calcium-dependent intracellular adhesion. They are the core of adhesion junctions and include many subtypes, such as E(epithelial)-, N(neutral)-, VE(vascular-endothelial)-, P(placental)-, R(retinal)-, and K(kidney)-cadherins [22]. E-cadherin is the primary mediator of intracellular adhesion in epithelial cells. The extracellular region of E-cadherin binds to other cadherins present on adjacent cells. Its intracellular part interacts with catenin molecules consisting of β-catenin, γ-catenin, and other regulatory proteins [23]. α-Catenin directly binds to the N-terminal region of β-catenin and thereby connects the cadherin-catenin complex to the actin filaments [24], which promotes adhesion junction proteins clustering and stabilizes the cell adhesion (Fig. 2). Without a Wnt stimulus, the majority of β-catenin locate at the membrane. It contributes to forming the E-cadherin/β-catenin complex, building an epithelial barrier, and restricting cell invasion and metastasis [25].

#### β-Catenin: transcriptional activator

**Fig. 3:**
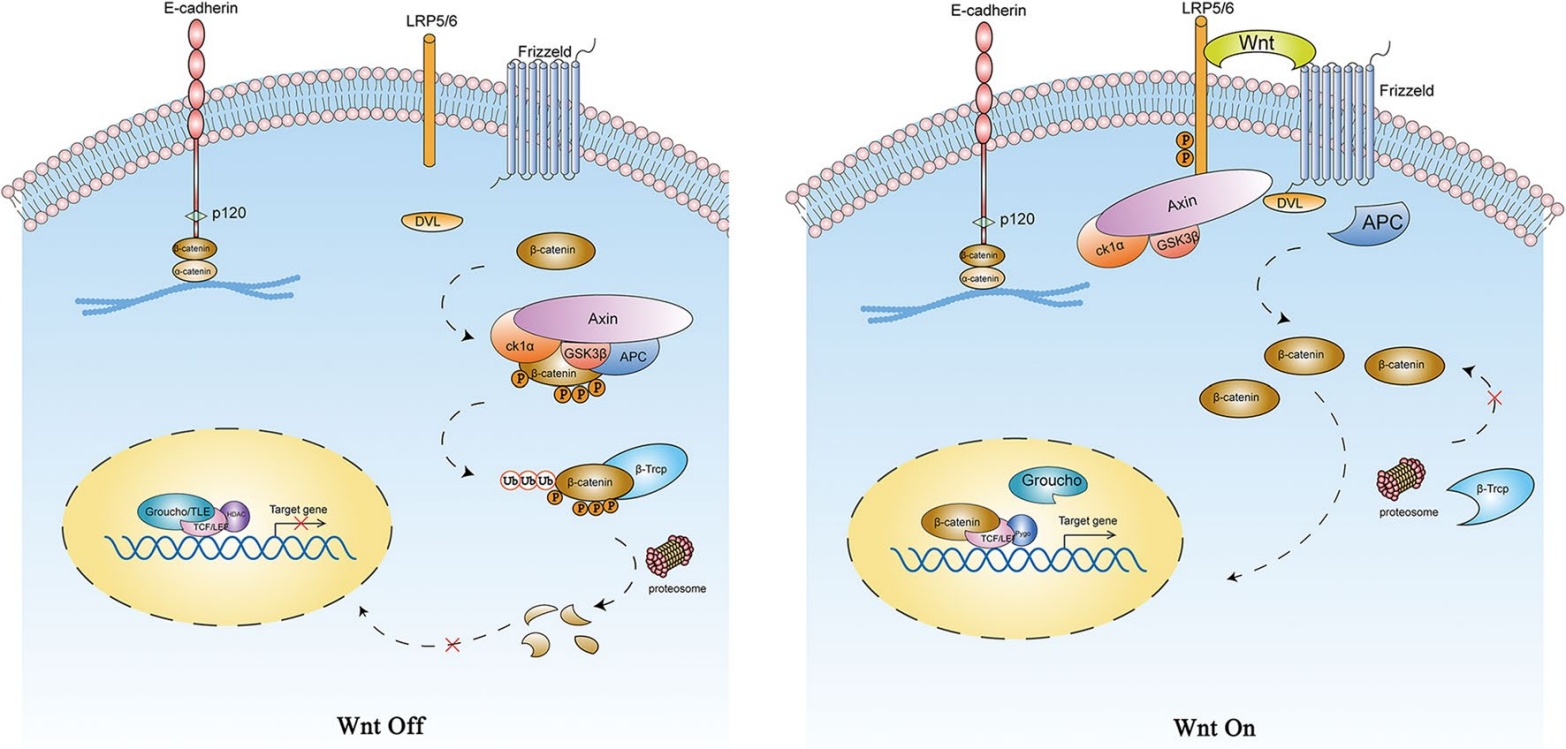
Wnt/β-catenin signaling pathway. In the absence of the Wnt signal, free cytoplasmic β-catenin is recognized and recruited by the destruction complex, where CK1 and GSK3β phosphorylate it, thereby undergoes ubiquitination-dependent proteolysis. In the presence of Wnt ligands, Wnt signaling blocks the activity of the destruction complex, and subsequently, β-catenin accumulate. Increased levels of cytoplasmic β-catenin translocate into the nucleus, where it associates with DNA binding proteins from the TCF/LEF family and other co-transcription factors. β-Catenin-TCF/LEF transcription complex then drives the transcription of Wnt/β-catenin target genes

β-Catenin is a pivotal component of the canonical Wnt signaling pathway, regulating embryonic development, tissue regeneration, stem cell maintenance, and adult tissue homeostasis [26]. In the canonical Wnt pathway, the Wnt signal is the chief regulator of β-catenin. It induces the stabilization of β-catenin in the cytoplasmic pool and initiates intracellular signaling via β-catenin nuclear translocation. In the Wnt “off” state, cytosolic β-catenin is tightly regulated by a so-called β-catenin destruction complex, which comprises scaffold protein Axin, tumor suppressor adenomatous polyposis coli (APC), two kinases casein kinase 1(CK1) and glycogen synthase kinase 3β(GSK3β) [27, 28]. Upon Axin and APC recognizing free β-catenin, CK1, and GSK3β induce amino-terminal serine-threonine phosphorylation of β-catenin [29], making β-catenin for ubiquitination dependent proteolysis [30]. In the Wnt “on” state, Wnt glycoproteins bind to Frizzled (FZD) and low-density lipoprotein receptor-related protein (LRP), thereby recruiting the disheveled (DVL) to the cell membrane [31]. Resultant FZD-LRP-DVL polymers then induce the docking of Axin and reassemble the destruction complex [32, 33]. β-Catenin accumulates in the cytoplasm and translocates into the nucleus where it displaces Groucho from TCF/LEF and activates the transcription of downstream genes such as cyclin-D1, c-Myc, Axin 2 (Fig. 3) [34].

Interestingly, accumulated evidence has verified that the disruption of the E-cadherin/β-catenin complex affects not only the intracellular adhesion but also the canonical Wnt pathway. Many target genes of Wnt canonical cascade, such as Twist and Slug, could also influence cadherin adhesion [35]. Therefore, cadherin loss and increased β-catenin signaling, which are attributed to imbalanced dual functions of β-catenin, can cooperate to induce both carcinoma and development.

### Regulation of β-catenin functions through phosphorylation

β-Catenin executes two main functions in epithelial cells. The degree to which β-catenin participates in both roles is regulated through phosphorylation by various kinases (Table 1). This phosphorylation regulatory mechanism modulates the binding affinity of β-catenin and promotes the controlled formation of protein–protein interactions [36]. Here, we only discuss the essential phosphorylation sites.

**Table 1:** Regulation mechanisms of β-catenin phosphorylation

Subcellular localization	Regulators	Sites	Function	Reference(s)
Membrane	Fer, Fyn, c-Met	Y142	Diminishes α-catenin binding	[38]
	c-Src,cMet, EGFR	Y654	Dissociates the E-cadherin/β-catenin complex	[44]
	Ab1	Y489	Dissociates the E-cadherin/β-catenin complex	[45]
	c-Src	Y86	Promotes the separation of Y654 and E-cadherins	[37]
	PKD1	T120	Transports β-catenin to the Golgi apparatus	[46]
	PT1B	Y654	Removes the phosphate group from the phosphorylated Y654 site	[47]
Cytoplasm	CK1	S45	Embarks β-catenin for β-Trcp recognition	[48]
	GSK3β	S33, S37, T41	Embarks β-catenin for β-Trcp recognition	[48]
Nucleus	Fer,Fyn,Yes,and Src	T142	Promotes β-catenin binding to the nuclear transporter Bcl9	[38]
	AKT and PKA	S552, S675	Strengthens β-catenin/TCF reporter activation	[42, 43]
	PKAL	S663	Regulates its transcriptional activity and induces target gene expression	[49]
	CK1	S45	Sox9 transfers the destruction complex “GSK3β-βTRCP-CK1-Axin” into the nucleus and diminishes nuclear β-catenin	[110]
	GSK3β	S33, S37, T41	Sox9 transfers the destruction complex “GSK3β-βTRCP-CK1-Axin” into the nucleus and diminishes nuclear β-catenin	[110]

#### Phosphorylation of β-catenin at the membrane

Phosphorylation of β-catenin at the membrane often leads to a weakening of the E-cadherin/β-catenin complex [37]. Two tyrosine residues in β-catenin have received the most attention: tyrosine 142 (Y142) in the first armadillo repeat and tyrosine 654 (Y654) in the last. Y142 is vital for α-catenin binding, and Y654 is crucial for E-cadherin. Phosphorylation at Y142 by the Fer, Fen, and c-Met tyrosine kinases diminish α-catenin binding [38]. It results in loss of binding to α-catenin and favors β-catenin binding to the nuclear transporter B-cell lymphoma-9 (Bcl9). Phosphorylation of Y654 by the epidermal growth factor receptor (EGFR) or Src inhibits the catenin/E-cadherin interaction. It gives rise to enhance phosphorylated β-catenin binding affinity for multiple transcription factors, for example, TATA-binding protein (TBP), CREB-binding protein (CBP), and TCF4 [39].

#### Phosphorylation of β-catenin in the cytoplasm

The significance of phosphorylation at Serine45(S45), Serine33(S33), Serine37(S37), and Threonine41(T41) sites is underscored for maintaining normal cytoplasmic β-catenin concentration. Without Wnt stimulation, β-catenin is recruited by the destruction complex. It is first phosphorylated at S45 by CK1 and then at S33, S37, and T41 by GSK3β [40]. S33 and S37 phosphorylation create a binding site for the β-transducin repeat-containing protein (β-TrCP) E3-ligase complex, leading to ubiquitination and rapid degradation by the 26S proteasome [41]. In contrast, Wnt signaling represses β-catenin phosphorylation, thus inducing β-catenin cytoplasmic accumulation and cytoplasmic-nuclear trafficking. Mutations at such S/T residues above might hamper β-catenin function and have been described in human colorectal cancer and several other malignancies.

#### Phosphorylation of β-catenin in the nucleus

β-Catenin does not possess any DNA-binding domain, so it functions by interacting with other DNA binding transcription co-factors. Previous findings demonstrated that phosphorylation of β-catenin might modulate the recruitment of these factors. β-Catenin Y142 phosphorylation by c-Met promotes Bcl9-2 binding, which acts as co-activators in Wnt signaling [39]. Protein kinase B (AKT) and Protein kinase A (PKA) phosphorylate β-catenin at residues S552 and S675 and strengthen β-catenin/TCF reporter activation, possibly through association with histone acetylase [42, 43]. Phosphorylation of β-catenin at S663 by PKAL regulates its transcriptional activity and induces target gene expression [43].

### Interaction between HPV and β-catenin

Unlike other cancers, persistent infection of HPV plays a unique role in cervical carcinogenesis. There are three events during HPV infection as cancer promoters: viral DNA integrates to host genome, viral oncoproteins (E6 and E7) express in the epithelial cells, and viral oncoproteins interact with cellular proteins [50]. The well-known oncogenic model is that E6/E7 binds and inactivates the tumor suppressor proteins p53 and retinoblastoma protein (pRb), respectively, interrupting cell-cycle control and initiating gene transcription [51]. E6 and E7 interact with a plethora of cellular proteins involved in cell signaling that participate in the establishment of the malignant phenotype, including β-catenin (Table 2).

**Table 2:** Interactions between HPV oncoproteins and β-catenin in Wnt signaling

HPV oncoproteins	Interactions	Biological effects
E6	E6-E6AP-β-catenin	Blocks proteasomal degradation of β-catenin and promotes its translocation to the nucleus
	E6-Dlg2/Scribble-APC-β-catenin	Inactivates APC and leads to β-catenin nuclear accumulation through E6-induced degradation of the Dlg1 and Scribble
	E6-MZF1/NKX2-1/FOXM1-β-catenin	Induces MZF1 expression and then activates NKX2-1 transcription, finally promotes FOXM1- mediated β-catenin nuclear translocation
	E6-p53-Siah-1/SIP/Ebi-β-catenin	E6-induced loss of p53 inactivates Siah-1/SIP/Ebi complex, which can act as β-TrCP and lead to β-catenin degradation
E7	E7-PP2A-β-catenin	Binds to both the catalytic and structural subunits of PP2A and consequently induces Wnt activation

Lichtig et al. demonstrated for the first time that HPV 16 E6 enhances β-catenin/TCF transcription in an E6 associated protein(E6AP)-dependent manner, providing a possible mechanistic link between HPV and the β-catenin signaling in cervical cancer cell lines [52]. Sominsky et al. proposed that E6/E6AP stimulates and augments Wnt signaling through different mechanisms. The Wnt-stimulatory function requires the activity of E6AP and GSK3β [53]. Recent investigations have made it evident that HPV E6 is related to the Forkhead box protein M1 (FOXM1), which interacts with β-catenin and activates the Wnt pathway in mammalian cells [54]. In E6-expressing cells, E6 induces Myeloid zinc finger 1 (MZF1) expression, and MZF1 activates NK2 homeobox 1 (NKX2-1) transcription. The promoter of FOXM1 harbors three putative sites for NKX2-1. Thus E6 upregulates FOXM1, activates the Wnt/β-catenin pathway, and initiates cancer carcinogenesis through the E6/MZF1/NKX2-1/FOXM1/β-catenin transcriptional factors axis [55]. We found part of the evidence in the interaction between E6 and proteins harboring PDZ domains. Disks large homolog 1(Dlg1) and Scribble contain PDZ domains and interact with APC. E6 targets Dlg1 and Scribble, leading to their degradation [52]. HPV-induced loss of p53 may contribute to aberrant β-catenin expression in cancers [56]. The ubiquitin ligase type 3 Siah-1 binds ubiquitin-conjugating enzymes and Ebi, acts as β-TrCP, and results in β-catenin degradation independent of the phosphorylation sites recognized by β-TrCP. P53 and DNA damage activate the Siah-1/SIP/Ebi-dependent pathway for β-catenin destruction. Other studies of GSK3β indicated that HPV 16 E6/E7 increases GSK3β transcripts through a region between 85 bp and 250 bp upstream of the transcription initiation site of human *GSK3β* gene, which in turn modulates cellular β-catenin [57]. The impact of the E7 in regulating Wnt signaling has not been studied as well as that of E6. In the double-transgenic mice model, co-expression of HPV 16 E7 and a constitutively active β-catenin could accelerate cervical carcinogenesis [58]. Protein phosphatase 2 (PP2A), a family of multimeric serine/threonine phosphatases, is also involved in the β-catenin degradation complex. PP2A directly interacts with APC and Axin, dephosphorylates S9 residue of GSK3β, and ultimately inhibits Wnt signaling. In the human keratinocytes model immortalized by HPV E6 and E7 oncoproteins and transformed with SV40 small T antigen (smt), Uren et al. discovered that smt directly inactivates PP2A, which leads to β-catenin accumulation [59]. Other findings suggested HPV 16 E7 binds to both the catalytic and structural subunits of PP2A and, consequently, induces Wnt activation [60].

With the previous evidence, HPV oncoproteins directly or indirectly interact with β-catenin, subsequently activate β-catenin signaling, and finally promote cervical carcinogenesis. Thus, HPV may be the initial hit in the multistep tumorigenesis, alterations of β-catenin and hyperactivation of the Wnt/β-catenin pathway in cervical cancer cells serve as a second hit to accomplish the development of cervical cancer.

### The oncogenic role of β-catenin in cervical carcinogenesis

#### The role of β-catenin in the initiation of cervical cancer

In various types of cancer, deregulation of the Wnt/β-catenin signaling pathway occurs almost invariably via mutations in *APC* gene, dysfunction of GSK3β, or mutations of β-catenin itself [61]. However, in cervical cancer tissue or cancer-derived cell lines, mutations in Wnt pathway members, such as *CTNNB1*, have rarely been detected [20, 62]. We posit that occasional variations in CTNNB1 accompany β-catenin abnormal expression in cervical cancer, but it is accomplished by employing upstream β-catenin regulators. Some proteins contribute to this process either directly or indirectly. In vitro and in vivo analyses showed that transcription factor AP-2β interacts with β-catenin through the DNA-binding domain of AP-2β and the 1-9 Armadillo repeats of β-catenin. AP-2β forms a complex with β-TrCP and β-catenin, thereby enhancing β-catenin degradation independent of the proteasome degradation system [63]. Li et al. confirmed that SRY-related HMG-box 17 (SOX17), a member of the SOX transcription factor family, binds to the region between − 1756 and − 1473 of β-catenin and trans-suppresses β-catenin in cervical cancer cells, resulting in downregulation of β-catenin target genes and inhibition of tumor formation [64]. The Dickkopf 3 (Dkk3) protein was significantly downregulated and showed a strong regulation of β-catenin in cervical cancer cells. Though Dkk3 indirectly binds to β-catenin, it could interact with β-TrCP, enhance the degradation of cytoplasmic β-catenin, and lead to its anti-proliferative activity [65]. Leucine-rich repeats containing G protein-coupled receptors 5 (LGR5) was also found to regulate β-catenin in human cervical cancer indirectly. The possible mechanism is that LGR5 recruits the LRP-FZD receptor complex and reinforces Wnt signaling following the phosphorylation of LRP, ultimately induces LGR5-mediated promotion of cervical cancer growth via the Wnt/β-catenin signaling pathway [66]. Li et al. suggested that Dosage-sensitive sex reversal, adrenal hypoplasia critical region, on chromosome X, gene 1 (DAX1) may bind to the site of −T222 to − 444 of GSK3β promoter and transcriptionally repress GSK3β in cervical cancer cell lines. Therefore, DAX1 promotes cancer cell growth and tumorigenicity by allowing β-catenin to escape degradation via GSK3β [67]. Wang et al. reported that the expression level of Na^+^/H^+^ exchanger regulating factor 1 (NHERF1) is reduced significantly in cervical cancer tissues. NHERF1 regulates β-catenin protein levels in HeLa and CaSki cells. It may attenuate β-catenin expression via suppression of α-actinin-4 (ACTN4) expression and inhibit cervical cancer cell proliferation via the NHERF1-ACTN4-β-catenin axis [68].

The roles of non-coding RNAs should also be valued. MicroRNAs (miRs) are a class of non-coding RNAs with ~ 20 to 22 nucleotides in length and negatively regulate the expression of various cancer-related genes by binding to 3′-untranslated regions (UTRs) [69]. Recent studies have suggested that miR-135a regulates β-catenin expression through abating Siah1 and possibly APC in the presence of high-risk HPV E6/E7 [70]. Wei et al. proposed that the overexpression of miR-638 inhibits the Wnt/β-catenin signaling pathway and correlates with the prognosis of cervical cancer patients [71]. Ji X’s team has demonstrated that miR-139 targets and inhibits TCF4, the critical transcription factor cooperates with β-catenin to activate the downstream target genes in response to Wnt signals. MiR-139 thereby significantly blocks β-catenin signaling and decreases cell proliferation in cervical cancer cells [72]. Li et al. reported that miR-378 targets the 3′-UTR of the suppression of tumorigenicity 7 like (ST7L) mRNA and affects the regulation of Wnt/β-catenin pathway [73]. ST7L is a tumor-suppressor gene and inactivates the Wnt/β-catenin pathway in various cancers [74]. Long non-coding RNAs (LncRNAs) are a class of single-stranded RNAs, more than 200 nucleotides (nt) long, which are incapable of coding proteins. LINC00675 expression causes a significant increase of β-catenin, and LINC00675 knock-down increases the phosphorylation of β-catenin, which causes degradation of β-catenin in the cytoplasm and nucleus. LINC00675 may affect Wnt/β-catenin signaling in cervical cancer and represent a potential diagnostic marker and therapeutic target for cervical cancer treatment [75]. The luciferase assay and western blot results revealed that highly expressed long non-coding RNA NNT-AS1 activates the β-catenin cascade and promotes cell proliferation in cervical cancer [76]. CASC11 is a newly discovered lncRNA located ~ 2.1 kb upstream of c-Myc in chromosome 8q24 gene desert. Hsu et al. indicated that CASC11 activates the Wnt signaling, upregulates the β-catenin, and promotes cervical cancer progression [77]. Other lncRNAs, for example, Bladder cancer-associated transcript 1 (BLACAT1), and Taurine upregulated 1 (TUG1), also supports cervical cancer by altering β-catenin and modulating the Wnt signaling pathway [76].

#### The role of β-catenin in the epithelial-mesenchymal transition of cervical cancer

As critical epithelial markers, the lack of β-catenin at the membrane promotes epithelial cells to convert to mesenchymal cells, which have been recognized as an essential process during carcinogenesis. Frequent cytoplasmic localization of β-catenin observed in cervical cancer tissue, and nuclear β-catenin expression was faint [78]. These results differ from those found for colorectal carcinoma [79], hepatocellular carcinoma [47], and prostatic cancer [80], β-catenin accumulates within the nucleus or cytoplasm. In a tissue microarray study of 147 cervical cancer cases, the impairment of E-cadherin and β-catenin expression is widespread in early-stage cervical cancers [81]. Jing et al. demonstrated that E-cadherin and β-catenin expression levels are gradually reduced with cervical cancer progression. The expression pattern of β-catenin differs on a molecular level, as the β-catenin protein level in the cell membrane declined, while its cytoplasmic expression level is upregulated [82]. Fujimoto J et al. have found that the integral expression of E-cadherin, α- and β-catenin mRNAs are suppressed in the metastatic lesions of advanced uterine cervical cancers. At the same time, it was not present in the primary tumors. Thus, the suppressed expression of main adhesion molecules might lead to invasiveness and metastatic potential of advanced uterine cervical cancers as one rate-limiting step [83]. Gregg et al. proposed that E-cadherin, α-, β-, and γ-catenin membrane location is decreased in squamous cell cancer compared to normal cervical epithelium. It’s associated with increased metastatic potential due to the loss of intercellular adhesion function and cell polarity related to epithelial-mesenchymal transition (EMT) [84]. A large number of experiments have verified the abnormal expression pattern of β-catenin in cervical cancer cells, so what are the factors that caused the ectopic expression of β-catenin? Experimental data confirmed that the Wnt antagonists Secreted Frizzled Related Protein 1 (SFRP1) and Wnt antagonists Secreted Frizzled Related Protein 2 (SFRP2) suppress the EMT of cervical cancer cells through Wnt signaling [85]. He et al. observed that S100A9 induces cell migration and EMT, while the S100A9-induced promotion of EMT significantly suppressed by downregulation of β-catenin [86]. Combined in vitro and in vivo research found that miR-4524b regulates the migration and invasion ability of cervical cancer by targeting Wilms’ tumor X protein (WTX), and WTX regulates the expression of β-catenin [87]. The inhibitor of β-catenin and TCF (ICAT) acts as a Wnt/β-catenin signaling pathway suppressor via blocking the binding of TCF to β-catenin. Jiang et al. confirmed that overexpressed ICAT promotes cervical cancer EMT by competing for the E-cadherin binding to β-catenin, thus disrupting the E-cadherin/β-catenin complex [88]. Zhang et al. indicated that cysteine-rich protein 1 (CRIP1) upregulates the mesenchymal markers vimentin and N-cadherin, elevates cytoplasmic β-catenin expression, and downregulates the epithelial molecule—E-cadherin. The present study revealed that CRIP1 is concerned with the aberrant Wnt/β-catenin signaling pathway and promotes migration and invasion of cervical cancer [89].

#### The role of β-catenin in chemo-/radio-therapy resistance

Primary or secondary resistance to chemotherapy or radiotherapy results in treatment failure. The molecular mechanisms involved in chemo-radiotherapy resistance are complex. Accumulating evidence has found that β-catenin may participate in these molecular pathways. Zhang et al. observed that the nuclear localization of β-catenin is a poor prognostic marker and correlated with chemo-/radio-resistance in cervical squamous cell cancer [90]. Zhou’s study uncovered an indispensable role of Fat mass and obesity-associated gene (FTO). Indeed, these parameters could regulate the expression of β-catenin by reducing m6A levels in its mRNA transcripts and enhance the chemo-radiotherapy resistance in cervical cancer [91]. The previous study conducted by Wang et al. showed that the expression of Peptidyl-prolyl cis/trans isomerase NIMA-interacting 1 (Pin 1) displays resistance to cisplatin in five cervical cell lines. They implied that Pin1 modulates chemo-resistance by upregulating FOXM1 expression and the Wnt/β-catenin signaling pathway involved [92]. Xu et al. suggested that chemotherapy activates β-catenin signaling in an eukaryotic initiation factor 4E (eIF4E)-dependent manner. Blocking of eIF4E or β-catenin sensitizes cervical cancer to chemotherapy [93]. Cytosolic phospholipase A2α (cPLA2α), a predominant source of arachidonic acid, is specifically upregulated in cervical cancer. The inhibition of cPLA2α significantly increases chemosensitivity in cervical cancer cells, and this process has been proved to act in a β-catenin-dependent manner. In addition to being directly associated with cervical cancer development, β-catenin also mediates cell resistance to drugs and reduces the therapeutic effect [94]. Therefore, targeting β-catenin may have therapeutic value in overcoming chemo-/radio- resistance in cervical cancer.

### Clinical value of β-catenin in cervical cancer therapy

In addition to being considered a potential prognostic biomarker, β-catenin is also a vital molecular-target in cervical cancer therapy. Imura et al. assessed the prognostic influence of β-catenin by immunohistochemistry in cervical cancer patients. In this multivariate analysis, the low pathologic stage (stages I and II) and preservation of β-catenin expression is independently favorable prognostic factors [95]. Liang et al. found a negative association between WIF1 expression and β-catenin in 196 cervical cancer patients, 39 cervical intraepithelial neoplasia (CIN) patients, and 41 normal cervical epithelium (NCE) subjects. β-Catenin serves as a poor prognostic marker for cervical cancer [96]. Jiang et al. examined the expression of the representative EMT markers (β-catenin and E-cadherin) in normal cervical tissue, CIN1, CIN2-3, and squamous cervical cancer and found the expression of β-catenin reduces gradually during the progression of cervical squamous cell lesions. Patients with squamous cervical cancer with lymph node metastasis, parametrial invasion, negative E-cadherin, and negative β-catenin expression has shorter overall survival and disease-free survival [82]. Besides, nuclear β-catenin expression is related to chemo-resistance and radio-resistance in cervical cancer [90].

Several approaches for targeting β-catenin, which include enzyme inhibitors, β-catenin/TCF antagonists, and transcriptional regulators, have been developed. For example, a small-molecule MSAB directly targets β-catenin at Armadillo repeat region 2 and inactivates oncogenic β-catenin signaling [97]. Tankyrase inhibitors, like XAV939 [98], K-756 [99], and WXL-8 [100], stimulate β-catenin degradation by stabilizing Axin. PFK115-584 and CFP049090 are examples that perturb the β-catenin/TCF complex in a dose-dependent but the non-selective manner [101]. The nonsteroidal anti-inflammatory drugs (aspirin and indomethacin) modulate TCF activity and attenuate the β-catenin signaling [102]. Additionally, other compounds (CRT3/iCRT5/iCRT14/BC21/BC23/HI-B1) have been reported to suppress tumor growth via inhibiting β-catenin-TCF4 interaction [103–106]. PRI-724 is a first-in-class small molecule antagonist that inhibits the interaction between β-catenin and its transcriptional co-activator CBP [107]. Some compounds mentioned above have already progressed to phase II oncology trials [108], but some are still under the discovery phase. Presently, no drugs, specifically inhibiting β-catenin, have been approved for the market [109]. Though β-catenin is a potentially druggable target, the possibility of adverse side effects cannot be ignored; it will require more detailed studies to address the key issue of the specificity of β-catenin inhibitor.

## Conclusion

To sum up, β-catenin is a kind of multifunctional protein-encoding by *CTNNB1*. In epithelial, β-catenin regulates epithelial cell growth and intracellular adhesion. In the Wnt signaling pathway, it is a crucial effector controlled by Wnt proteins and modulates transcription of genes. The Wnt signaling and phosphorylation systems regulate the switch between β-catenin’s adhesive and nuclear translocation functions. Different forms (phosphorylated vs. non-phosphorylated) have different effects on carcinogenesis and tumor progression. It has become definite that β-catenin contributes to cervical cancer through accumulating experimental evidence. It has effects on promoting cell proliferation, mediating EMT, enhancing cell resistance to chemo-/radio- therapy. The alterations of β-catenin itself, disruption of E-cadherin/β-catenin complex, and molecules directly or indirectly impacting the abnormal expression of β-catenin may contribute to this progression. A more accurate perception of the β-catenin precise regulatory mechanism in cervical cancer carcinogenesis is still needed to conduct in the future. For instance, further studies could focus on the genetic mutations and epigenetic alterations of CTNNB1 in cervical cancer, the relationship between other HR-HPV oncoproteins or different HPV subtypes, and β-catenin, the precise biomolecules-β-catenin binding mechanisms. β-Catenin is a potential prognosis biomarker and a druggable target for cancer therapy. More profound research of β-catenin useful for diagnosis, treatment, and prognosis of cervical cancer in larger samples and multicenter settings is worth exploring.

## Data Availability

Not applicable.

## Abbreviations

HPV: human papillomavirus
TCF/LEF: T cell factor/lymphoid enhancer factor
ARM: Armadillo
NTD: amino terminus domain
CTD: carboxyl terminus domain
APC: adenomatous polyposis coli
CK1: casein kinase 1
GSK3β: glycogen synthase kinase 3β
FZD: Frizzled
LRP5/6: lipoprotein receptor-related protein 5/6
DVL: Disheveled
Y: tyrosine
Bcl9: B cell lymphoma-9
EGFR: epidermal growth factor receptor
TBP: TATA-binding protein
CBP: CREB-binding protein
S: serine
T: threonine
β-TrCP: β-transducin repeat-containing protein
PKA: protein kinase A
AKT: protein kinase B
E6AP: E6 associated protein
FOXM1: Forkhead box protein M1
MZF1: myeloid zinc finger 1
NKX2-1: NK2 homeobox 1
Dlg1: disks large homolog 1
PP2A: protein phosphatase 2
SOX17: SRY-related HMG-box 17
Dkk3: Dickkopf 3
LGR5: Leucine-rich repeats containing G protein-coupled receptors 5
DAX1: dosage-sensitive sex reversal, adrenal hypoplasia critical region, on chromosome X, gene 1
NHERF1: Na+/H+ exchanger regulating factor 1
ACTN4: α-actinin-4
UTR: untranslated regions
miRs: microRNAs
LncRNAs: long-non-coding RNAs
BLACAT1: bladder cancer-associated transcript 1
TUG1: Taurine up-regulated 1
EMT: epithelial–mesenchymal transition
SFRP1/2: Secreted Frizzled Related Protein 1/2
WTX: Wilms’ tumor X protein
ICAT: inhibitor of β-catenin and TCF
CRIP1: cysteine-rich protein 1
FTO: fat mass and obesity-associated gene
PIN1: peptidyl-prolyl cis/trans isomerase NIMA-interacting 1
eIF4E: eukaryotic initiation factor 4E
CIN: intraepithelial neoplasia
NCE: normal cervical epithelium
